# RETRACTED: Das et al. Carnosic Acid Attenuates Cadmium Induced Nephrotoxicity by Inhibiting Oxidative Stress, Promoting Nrf2/HO-1 Signalling and Impairing TGF-β1/Smad/Collagen IV Signalling. *Molecules* 2019, *24*, 4176

**DOI:** 10.3390/molecules31183312

**Published:** 2026-09-18

**Authors:** Sonjit Das, Saikat Dewanjee, Tarun K. Dua, Swarnalata Joardar, Pratik Chakraborty, Shovonlal Bhowmick, Achintya Saha, Simanta Bhattacharjee, Vincenzo De Feo

**Affiliations:** 1 Advanced Pharmacognosy Research Laboratory, Department of Pharmaceutical Technology, Jadavpur University, Kolkata 700032, India; dsonjit@gmail.com (S.D.); tarunkduaju@gmail.com (T.K.D.); swarnalatajoardar@yahoo.in (S.J.); pratik.chakraborty88@yahoo.com (P.C.); contact.simanta@gmail.com (S.B.); 2 Department of Chemical Technology, University of Calcutta, Kolkata 700009, India; sovonlal@gmail.com (S.B.); achintya_saha@yahoo.com (A.S.); 3 Department of Pharmacy, University of Salerno, 84084 Fisciano, Italy

The journal retracts the article titled “Carnosic Acid Attenuates Cadmium Induced Nephrotoxicity by Inhibiting Oxidative Stress, Promoting Nrf2/HO-1 Signalling and Impairing TGF-β1/Smad/Collagen IV Signalling” [1], cited above.

Following publication, concerns were brought to the attention of the Editorial Office regarding the integrity of a number of figures presented in this article [1].

In accordance with the journal’s standard procedures, the Editorial Office and Editorial Board conducted an investigation that identified indications of inappropriate image editing and apparent duplications within Figures 3, 4, 7, and 9, as well as indications of duplication between Figure 1A presented in this article [1] and images from earlier publications [2,3] produced by similar authorship groups, reported as originating from different experiments. Although the authors cooperated during the investigation, the Editorial Board concluded that the explanations and the supporting materials provided could not satisfactorily resolve the identified concerns. 

As a result, the Editorial Board has lost confidence in the reliability of the findings reported in this article and has decided to retract the publication as per MDPI’s retraction policy.

This retraction was approved by the Editor-in-Chief of the journal *Molecules*. 

The authors have been informed of this retraction and have indicated their disagreement with this decision.
